# Use of Inhaled Epoprostenol in Patients With COVID-19 Receiving Humidified, High-Flow Nasal Oxygen Is Associated With Progressive Respiratory Failure

**DOI:** 10.1016/j.chstcc.2023.100019

**Published:** 2023-09-25

**Authors:** Andrew P. Michelson, Patrick G. Lyons, Nguyet M. Nguyen, Daniel Reynolds, Rachel McDonald, Colleen A. McEvoy, Vladimir Despotovic, Steven L. Brody, Marin H. Kollef, Bryan D. Kraft

**Affiliations:** Division of Pulmonary and Critical Care Medicine, Washington University School of Medicine, Saint Louis, MO.; Department of Medicine, the Institute for Informatics, Washington University School of Medicine, Saint Louis, MO.; Division of Pulmonary and Critical Care Medicine, Washington University School of Medicine, Saint Louis, MO.; Division of Pulmonary and Critical Care Medicine, Washington University School of Medicine, Saint Louis, MO.; Division of Pulmonary and Critical Care Medicine, Washington University School of Medicine, Saint Louis, MO.; Division of Pulmonary and Critical Care Medicine, Washington University School of Medicine, Saint Louis, MO.; Division of Pulmonary and Critical Care Medicine, Washington University School of Medicine, Saint Louis, MO.; Division of Pulmonary and Critical Care Medicine, Washington University School of Medicine, Saint Louis, MO.; Division of Pulmonary and Critical Care Medicine, Washington University School of Medicine, Saint Louis, MO.; Division of Pulmonary and Critical Care Medicine, Washington University School of Medicine, Saint Louis, MO.; Division of Pulmonary and Critical Care Medicine, Washington University School of Medicine, Saint Louis, MO.

**Keywords:** Acute respiratory distress syndrome, ARDS, COVID-19, humidified high-flow nasal cannula, inhaled epoprostenol, inhaled pulmonary vasodilators, SARS-CoV-2

## Abstract

**BACKGROUND:**

The clinical benefit of using inhaled epoprostenol (iEpo) through a humidified high-flow nasal cannula (HHFNC) remains unknown for patients with COVID-19.

**RESEARCH QUESTION:**

Can iEpo prevent respiratory deterioration for patients with positive SARS-CoV-2 findings receiving HHFNC?

**STUDY DESIGN AND METHODS:**

This multicenter retrospective cohort analysis included patients aged 18 years or older with COVID-19 pneumonia who required HHFNC treatment. Patients who received iEpo were propensity score matched to patients who did not receive iEpo. The primary outcome was time to mechanical ventilation or death without mechanical ventilation and was assessed using Kaplan-Meier curves and Cox proportional hazard ratios. The effects of residual confounding were assessed using a multilevel analysis, and a secondary analysis adjusted for outcome propensity also was performed in a multivariable model that included the entire (unmatched) patient cohort.

**RESULTS:**

Among 954 patients with positive SARS-CoV-2 findings receiving HHFNC therapy, 133 patients (13.9%) received iEpo. After propensity score matching, the median number of days until the composite outcome was similar between treatment groups (iEpo: 5.0 days [interquartile range, 2.0-10.0 days] vs no-iEpo: 6.5 days [interquartile range, 2.0-11.0 days]; *P* = .26), but patients who received iEpo were more likely to meet the composite outcome in the propensity score-matched, multilevel, and multivariable unmatched analyses (hazard ratio, 2.08 [95% CI, 1.73-2.50]; OR, 4.72 [95% CI, 3.01-7.41]; and OR, 1.35 [95% CI, 1.23-1.49]; respectively).

**INTERPRETATION:**

In patients with COVID-19 receiving HHFNC therapy, use of iEpo was associated with the need for invasive mechanical ventilation.

Inhaled prostacyclins are an attractive adjunctive therapy for the treatment of ARDS because of their ability to reduce hypoxia-induced vasoconstriction, to improve ventilation-perfusion matching, and to prevent platelet-mediated thrombosis.^1,2^ When delivered to patients with ARDS via tracheal tube, clinical parameters, such as Pao_2_, Pao_2_ to Fio_2_ ratio, and mean pulmonary artery pressure can improve significantly.^1,3^ Despite improvement in these physiologic parameters, studies evaluating the use of inhaled prostacyclins failed to show a reduction in mortality or time receiving mechanical ventilation.^1,2,4-7^ As a result, the use of inhaled pulmonary vasodilators remains adjunctive for ARDS and currently is not recommended by most critical care-related society guidelines.^8-10^

During the COVID-19 pandemic, use of oxygen delivered by humidified high-flow nasal cannula (HHFNC) for SARS-CoV-2-induced ARDS has increased.^11,12^ As a result, many clinicians have considered extending the diagnostic criteria of ARDS to include the use of HHFNC in an attempt to identify patients earlier and to facilitate enrollment in research studies.^13,14^ Use of inhaled pulmonary vasodilators through the HHFNC circuit also has proven to be technically feasible and safe, although with inconsistent effects on gas exchange.^6,15-18^ Nevertheless, the ability of inhaled prostacyclins to prevent progression to invasive mechanical ventilation for patients with SARS-CoV-2-induced ARDS receiving HHFNC therapy has not been well evaluated.^6,16-18^ Therefore, the objective of this study was to determine if inhaled epoprostenol (iEpo) delivered through HHFNC was associated with a reduced rate of respiratory failure progression in patients with SARS-CoV-2 pneumonia.

## Study Deign and Methods

### Study Population

This retrospective study included patients admitted to 13 hospitals of the BJC HealthCare System between March 1, 2020, and September 23, 2021. This system includes one academic medical center and 12 community hospitals. The Washington University School of Medicine Institutional Review Board approved this study with a waiver of informed consent (Identifier: 202003122).

All patients aged 18 years or older with a positive finding for SARS-CoV-2 on a polymerase chain reaction or antigen test in the 14 days before or 7 days after admission and who were started on HHFNC therapy were included for analysis.^19^ Patients were excluded if they were intubated and mechanically ventilated before HHFNC therapy was initiated during the same encounter or had a limited resuscitation order or comfort care order placed after HHFNC therapy initiation. Patients who received inhaled nitric oxide were excluded from this study. Only the encounter associated with the patient’s first positive COVID-19 test results was evaluated.

### Data Collection and Preprocessing

Data for this analysis was extracted from the institutional COVID-19 DataMart, which contains electronic health record data including demographic, laboratory, vital sign, medication, hospital location, comorbidity, and procedure information. Clinical features with > 50% missingness were dropped and the remainder were median imputed. Sequential Organ Failure Assessment scores were calculated hourly using a last observation carried forward approach for 72 h before initiation of HHFNC therapy, and the median value was obtained for the 24 h preceding the time of HHFNC therapy initiation.^20^ If arterial blood sampling was not available within the lookback window, the ratio of peripheral oxygen saturation to Fio_2_ was used to estimate the Pao_2_ to Fio_2_ ratio.^20^

### Outcomes

The primary outcome was time from HHFNC therapy initiation to the earlier of intubation or death (because cardiac arrest without return of spontaneous circulation represents a competing risk for intubation with mechanical ventilation) by day 28. Patients discharged before day 29 were censored at the time of discharge. Patients who had not been ventilated invasively by day 28 were assumed to not have experienced the outcome.

### Statistical Analysis

#### Overview:

To determine if the use of iEpo was associated with progressive respiratory failure, patients with positive SARS-CoV-2 findings started on HHFNC and iEpo therapy were propensity score matched 1:2 with those receiving HHFNC therapy, but not iEpo. Time from HHFNC therapy initiation to the composite outcome was assessed using Kaplan-Meier curves and Cox proportional hazards. Because of an expected small sample size, we did not perform any sample size calculation a priori, but rather used all available patients at the time the analyses were designed.

#### Propensity Score Matching:

Propensity score matching was performed at the time of HHFNC therapy initiation at a ratio of 1:2. PsmPy, a propensity score matching algorithm released for Python (Python Software Foundation), was used to calculate propensity scores and to match patients using clinical characteristics available before HHFNC therapy initiation.^21^ Propensity scores were calculated using a balanced logistic regression, and the one-to-many matching algorithm was called to match on the calculated propensity logit score at a ratio of 1:2. Time-invariant characteristics were age, sex, race (Black, White, other), BMI, Elixhauser comorbidity score, tobacco use (never, unknown, quit, active), and receipt of any of the following medications: corticosteroids, remdesivir, baricitinib, or tocilizumab (modeled in a binary fashion). The Elixhauser comorbidity score was chosen because it contains more comorbidities than the Charlson comorbidity score, which may help with more granular propensity score matching. Time-variant characteristics included laboratory data (median values of WBC count, absolute lymphocyte count, hemoglobin, platelet count, creatinine, total bilirubin, albumin, D-dimer, and C-reactive protein), vital sign data (median of temperature, heart rate, respiratory rate, and mean arterial pressure), and severity of illness measures (Sequential Organ Failure Assessment score). Only time-varying data collected in the 24 h preceding HHFNC therapy initiation were included. To account for treatment variability that arose as a function of time, the study period was divided into sequential 180-day blocks from March 20, 2020. The block of time during which an individual treatment period occurred was used as a categorical variable in multivariable propensity score calculation for matching. To control for confounders associated with treatment location, we attempted to match based on hospital where the patient sought treatment. However, because of sample size constraints, we were unable to generate adequate propensity score matches based on individual hospitals or type of hospital (academic vs community). Matching performance was assessed by standard comparisons between the exposed cohort and the matched cohort, including calculation of standardized mean absolute error. All included variables had a standardized mean difference of < 0.2 (e-Figs 1-3).

#### Multilevel Analysis:

All patients matched in a 1:2 ratio were assigned to a relational group that included all patients in that match (e-Appendix 1). Noncategorical data were standardized. For categorical data, one item from each category was removed (example: for patient sex, female was retained and male was removed). The model was fit using the statsmodels *mixedlm* function, with the groups set to the relational group of the propensity score matching process. All variables used in the propensity score matching were included in the multilevel model, with a random effect for the relational group.

#### Sensitivity Analysis:

The risk for the composite outcome, mechanical ventilation, or death without mechanical ventilation across the entire, unmatched population was assessed using a logistic regression model with the same clinical variables with the addition of the propensity logit score.

#### Data Analysis:

Where appropriate, χ^2^ and Mann-Whitney *U* tests were performed. A *P* value of < .01 was considered statistically significant. All analyses were carried out in Python version 3.8.8 (Python Software Foundation) using the following packages: scipy, numpy, pandas, matplotlib, seaborn, PsmPy, and statsmodels.

## Results

### Patient Characteristics and Outcomes

**Figure 1 –:**
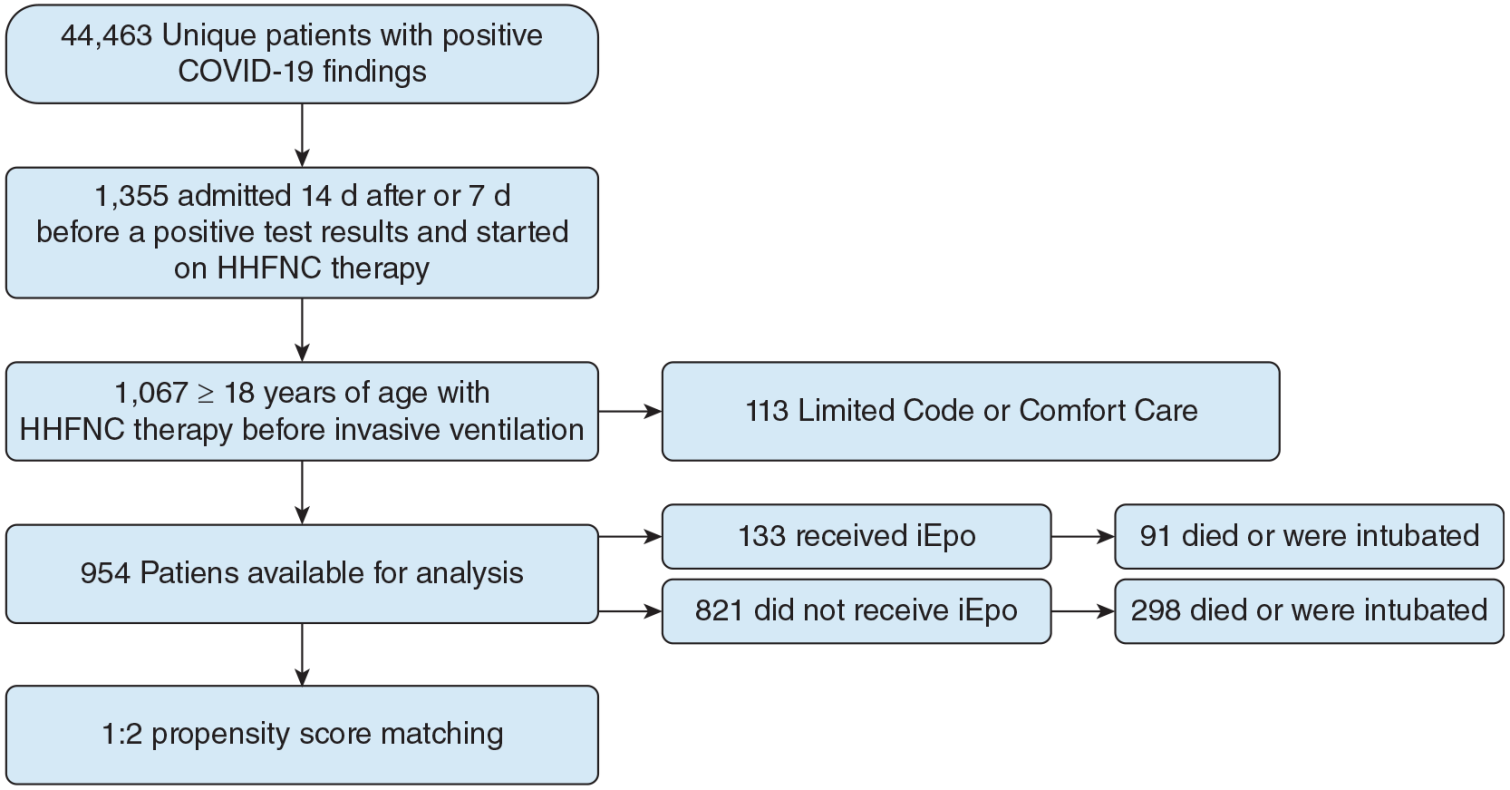
Flow diagram showing patient cohort. HHFNC = high-humidity nasal cannula; iEpo = inhaled epoprostenol.

Of the 44,463 patients with a positive finding for SARS-CoV-2 infection, 1,067 received HHFNC therapy before mechanical ventilation and 113 were excluded because of a limited resuscitation order placed after HHFNC therapy initiation. The remaining 954 patients were analyzed, and of those, 133 received iEpo (Fig 1) and one patient without a limited resuscitation order died before invasive mechanical ventilation was initiated. One hundred thirteen patients (85%) who received iEpo initially were admitted to an academic medical center. Two patients received inhaled nitric oxide and were excluded upfront. The median time to iEpo administration from HHFNC initiation was 1.03 days (interquartile range [IQR], 0.29-3.55 days) and the median duration of iEpo exposure before the primary outcome was 1.79 days (IQR, 0.39-3.51 days). After propensity score matching, all 133 patients who received iEpo were matched to 266 patients who did not receive iEpo for the primary analysis (Table 1). After matching, patients who received iEpo were similar to patients who did not receive iEpo (Table 1, e-Figs 1-3).

**Figure 2 –:**
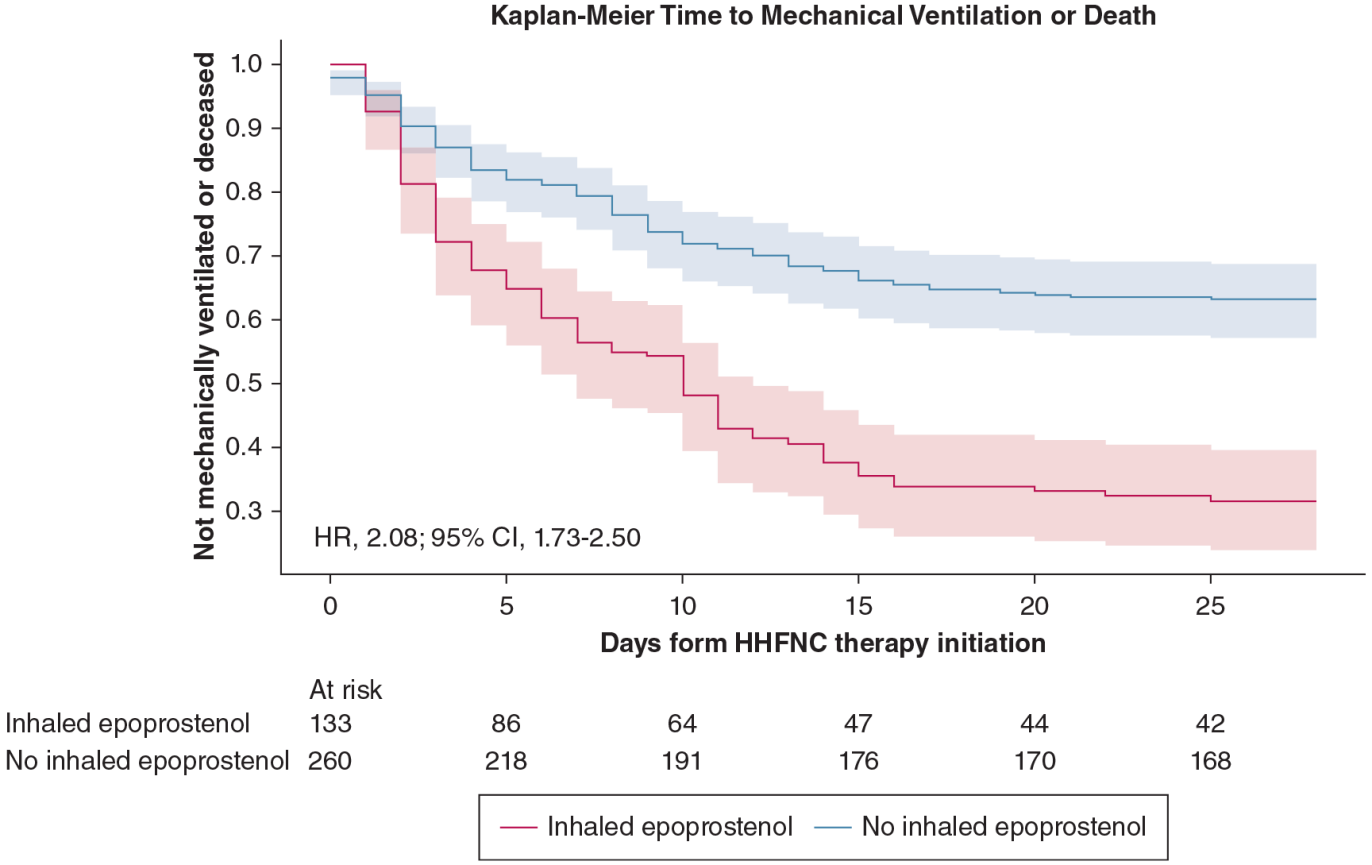
Kaplan-Meier curve depicting time to the composite outcome of time to mechanical ventilation or death without mechanical ventilation for patients initiated on HHFNC therapy who received inhaled epoprostenol as well as the matched control participants who did not receive inhaled epoprostenol. HHFNC = humidified high-flow nasal cannula; HR = hazard ratio.

In the matched cohort, 91 patients (68.4%) who received iEpo met the composite outcome within 28 days vs 99 patients (37.2%; *P* < .01) who did not receive iEpo. The time to mechanical ventilation or death without mechanical ventilation was similar between the groups (5.0 days [IQR, 2.0-10.0 days] vs 6.5 days [IQR, 2.0-11.0 days]; *P* = .26). In the primary analysis, use of iEpo was associated with a significantly higher risk of mechanical ventilation or death without mechanical ventilation (hazard ratio, 2.08; 95% CI, 1.73-2.50) (Fig 2).

To exclude significant residual confounding among measured variables as the cause for the differences observed in the matched cohort, we next performed a multilevel analysis among propensity score-matched participants. The association of iEpo with mechanical ventilation or death without mechanical ventilation remained statistically significant (OR, 1.35; 95% CI, 1.23-1.49) (e-Table 1).

### Sensitivity Analysis

Among the entire (unmatched) population, use of iEpo was associated significantly with progression to the composite outcome (OR, 4.72; 95% CI, 3.01-7.41) (Table 2).

## Discussion

In this retrospective analysis, the use of iEpo for patients receiving HHFNC therapy was associated significantly with an increased risk for the composite outcome of mechanical ventilation or death without mechanical ventilation, and this association persisted after multilevel modeling in a propensity score-matched cohort and multivariable modeling on an expanded, non-propensity score-matched population. This is one of the largest and most comprehensive studies to date assessing the clinical usefulness of inhaled pulmonary vasodilators in nonintubated patients and adds to the growing body of literature surrounding optimal management of acute hypoxemic respiratory failure for patients infected with SARS-CoV-2.

To date, no randomized controlled clinical trials have assessed the clinical impact of inhaled pulmonary vasodilators on mortality or days free of mechanical ventilation. Nevertheless, several studies have assessed the impact of inhaled pulmonary vasodilators on physiologic outcomes and have demonstrated that their use is associated with an improved Pao_2_ to Fio_2_ ratio, diastolic dysfunction, left ventricular end diastolic pressure, and pulmonary artery pressure.^1-5^ Although other studies have shown a less robust physiologic response, none have shown a clear worsening of physiologic parameters or have raised concerns about safety.^1-5,15,16,22^ Despite the potential short-term physiologic benefits of iEpo, in this study, the use of iEpo was associated with worse outcomes in both the primary analysis and related sensitivity analysis.

Previously, a similar report investigated the impact of iEpo through HHFNC administration for COVID-19-associated ARDS.^6^ In that study, the investigators noted that the time to invasive mechanical ventilation was longer in patients treated with iEpo compared with those not treated with iEpo, but the overall rate of progression to mechanical ventilation was the same.^6^ With 60 patients from a single center, adjustment for only a few parameters was feasible. This multicenter analysis overcomes some of the prior limitations by analyzing a larger population of patients and adjusting for multiple variables, thereby adding confidence to the finding that time to mechanical ventilation was approximately the same after propensity score matching. Further, we found that the rate of progression to invasive ventilation was significantly higher for patients receiving iEpo.

The principal cause for progressive respiratory failure associated with iEpo use remains unknown, although several mechanisms are possible. iEpo is a synthetic prostaglandin I2 molecule with potent immunomodulatory, vasodilatory, vascular permeability, and antithrombotic properties.^23-28^ It is possible that use of iEpo changes the host response to SARS-CoV-2 infection through these channels to impair lung healing or to exacerbate the ongoing injury. At the same time, it is also plausible that the antithrombotic effects of iEpo promote microvascular hemorrhage and further exacerbate impairment of gas exchange and parenchymal damage. Finally, although the time to mechanical ventilation may be a factor associated with poor outcomes, trials of HHFNC therapy before invasive mechanical ventilation have been shown to be safe, and in this study, the time to mechanical ventilation was the same between treatment groups, suggesting that optimal therapy was not delayed preferentially between treatment groups.^11,12,29^

This study has several limitations. First, our results are vulnerable to immortal time bias during the period from HHFNC therapy initiation to iEpo initiation. This period was relatively brief for most patients (median time from HHFNC initiation to iEpo administration was 1.03 days [IQR, 0.29-3.55 days]), especially as compared with the time from HHFNC initiation to outcomes for the control participants (median, 6.5 days [IQR, 2.0-11.0 days]). Moreover, to the extent that it is present, an immortal time bias would have conferred a false survival advantage in favor of iEpo treatment. Despite this influence, our study still showed that exposure to iEpo was associated significantly with progression to mechanical ventilation or death. Second, the results could be impacted by unmeasured differences in the processes of care between hospital sites (academic and community hospitals) and providers.^11^ Although we attempted to control for admission to a community vs academic hospital, our population size was not robust enough to perform adequate matching. As a result, residual confounding could be present, which arises because iEpo is not a standard component of ARDS management, so the timing and selection of patients could vary across health care delivery sites and providers; however, our multilevel analysis suggested that this alone may not be sufficient to account for the results measured in this analysis. Similarly, although the dosing for iEpo remains relatively standardized at a fixed dose of 16 ng/kg/min, treatment teams can modify the initial dosing and weaning parameters, which also could introduce heterogeneity into the study. Unfortunately, estimation of total iEpo dose received was not possible, but the impact of all these process-of-care variables likely was mitigated by the system-wide release of standardized treatment protocols, the use of tele-ICU services within community hospitals and by controlling for the use of advanced therapeutics, such as IL-6 inhibitors, within the model. Although adherence to this protocol was not measured directly, the use of medical components of care, as mentioned previously, were similar between treatment groups after propensity score matching (Table 1).

Third, although we attempted to account for alveolar drug delivery in our propensity scores by including the Pao_2_ to Fio_2_ ratio in the matching process, this measure may be an inadequate surrogate for delivery of aerosolized (as opposed to gaseous, eg, nitric oxide) therapeutics. Fourth, this study did not account for vaccination status because these data were not available in the data set. Fifth, it is also a retrospective analysis restricted to adults in the American Midwest and may not be applicable to other regions. Sixth, unmeasured confounding is likely; for instance, we were unable to account for cumulative fluid status, which has been shown to impact ARDS outcomes.^30,31^ Finally, this study was unable to account for potential superinfection, although we controlled for markers associated with infection (eg, temperature, respiratory rate, heart rate, WBC count) and the use of immunomodulators, which are contraindicated during active infection.

## Interpretation

This retrospective cohort study advances our knowledge about the use of iEpo in patients with COVID-19 ARDS receiving HHFNC therapy, finding that use of iEpo is associated with increased risk of mechanical ventilation or death without mechanical ventilation.

## Supplementary Material

1

## Figures and Tables

**TABLE 1:** Population Characteristics

Characteristic	Before Matching		After Matching	
	iEpo	No iEpo	iEpo	No iEpo
Demographics				
Population size	133 (13.9)	821 (86.1)^a^	133 (33.3)	266 (66.7)^a^
Age	61.0 (55.0-68.0)	67.0 (58.0-77.0)^a^	61.0 (55.0-68.0)	61.0 (51.0-70.8)
Female sex	57 (42.9)	352 (42.9)	57 (42.9)	122 (45.9)
Race				
Black	63 (47.4)	252 (30.7)^a^	63 (47.4)	146 (54.9)
White	56 (42.1)	544 (66.3)^a^	56 (42.1)	104 (39.1)
Other	14 (10.5)	25 (3.0)^a^	14 (10.5)	16 (6.0)
BMI	33.5 (28.1-40.4)	31.4 (26.8-37.9)^a^	33.5 (28.1-40.4)	34.2 (27.9-41.7)
Tobacco use				
Current	6 (4.5)	77 (9.4)	6 (4.5)	16 (6.0)
Former	52 (39.1)	342 (41.7)	52 (39.1)	143 (53.8)
Never	70 (52.6)	374 (45.6)	70 (52.6)	98 (36.8)
Unknown	5 (3.8)	28 (3.4)	5 (3.8)	9 (3.4)
Elixhauser score	9.0 (0.0-21.0)	12.0 (2.0-25.0)	9.0 (0.0-21.0)	9.0 (0.0-18.8)
SOFA score	2.0 (2.0-4.0)	3.0 (2.0-4.0)	2.0 (2.0-4.0)	3.0 (2.0-4.0)
Pao_2_ to Fio_2_ ratio	171.5 (122.2-244.0)	183.0 (130.0-236.0)	171.5 (122.2-244.0)	173.0 (128.0-212.5)
Laboratory results				
WBC count	8.1 (5.6-11.0)	8.3 (6.0-11.6)	8.1 (5.6-11.0)	7.8 (5.5-11.5)
ALC	0.8 (0.5-1.0)	0.8 (0.5-1.2)	0.8 (0.5-1.0)	0.9 (0.6-1.2)
Hemoglobin	12.7 (11.2-14.1)	12.5 (11.0-14.0)	12.7 (11.2-14.1)	12.6 (11.2-14.1)
Platelet count	203.0 (154.1-254.8)	214.0 (160.0-279.0)	203.0 (154.1-254.8)	194.5 (151.2-251.9)
Creatinine	1.0 (0.8-1.5)	1.0 (0.8-1.4)	1.0 (0.8-1.5)	1.0 (0.8-1.4)
Albumin	3.3 (3.1-3.5)	3.2 (2.9-3.5)	3.3 (3.1-3.5)	3.3 (3.0-3.6)
Total bilirubin	0.4 (0.3-0.6)	0.5 (0.3-0.7)	0.4 (0.3-0.6)	0.4 (0.3-0.6)
ALT	54.0 (43.2-70.4)	48.0 (31.0-75.0)	54.0 (43.2-70.4)	50.0 (34.5-71.2)
Vital signs				
GCS score	15.0 (15.0-15.0)	15.0 (15.0-15.0)^a^	15.0 (15.0-15.0)	15.0 (15.0-15.0)
Heart rate	88.0 (77.8-100.0)	84.0 (74.0-97.0)	88.0 (77.8-100.0)	86.8 (74.5-98.5)
MAP	93.7 (85.8-103.0)	91.3 (84.0-99.3)	93.7 (85.8-103.0)	92.3 (84.5-99.3)
RR	25.0 (22.0-29.5)	23.0 (20.0-27.0)^a^	25.0 (22.0-29.5)	24.0 (20.0-29.0)
Systolic BP	130.0 (120.0-146.0)	129.0 (118.0-141.0)	130.0 (120.0-146.0)	129.0 (118.0-139.0)
Spo_2_	93.0 (91.5-94.5)	93.0 (91.5-95.0)	93.0 (91.5-94.5)	93.0 (91.0-94.0)
Temperature	36.9 (36.7-37.3)	36.8 (36.6-37.1)	36.9 (36.7-37.3)	36.8 (36.6-37.1)
Medications				
Steroids	129 (97.0)	756 (92.1)	129 (97.0)	257 (96.6)
Remdesevir	118 (88.7)	692 (84.3)	118 (88.7)	233 (87.6)
Baricitinib	10 (7.5)	76 (9.3)	10 (7.5)	16 (6.0)
Tocilizumab	29 (21.8)	98 (11.9)^a^	29 (21.8)	53 (19.9)
Outcomes				
Ventilated	83 (62.4)	204 (24.8)^a^	83 (62.4)	80 (30.1)^a^
Time to ventilator, d	2.0 (1.0-6.0)	2.0 (0.0-6.0)	2.0 (1.0-6.0)	2.0 (0.0-6.2)
28-d mortality	54 (40.6)	186 (22.7)^a^	54 (40.6)	51 (19.2)^a^
Ventilator or death without ventilator	91 (68.4)	298 (36.3)^a^	91 (68.4)	99 (37.2)^a^
Time to ventilator or death, d	5.0 (2.0-10.0)	7.0 (3.0-12.0)	5.0 (2.0-10.0)	6.5 (2.0-11.0)

Data are presented as No. (%) or median (interquartile range). ALC = absolute lymphocyte count; ALT = alanine aminotransferase; iEpo = inhaled epoprostenol; GCS = Glasgow coma scale; MAP = mean arterial pressure; RR = respiratory rate; SOFA = Sequential Organ Failure Assessment; Spo_2_ = peripheral oxygen saturation by pulse oximetry.

a *P* < .01 between patients receiving iEpo and not receiving iEpo within the same match group.

**TABLE 2:** Independent Factors Associated With Progression of Respiratory Failure

Variable	Adjusted OR	95% CI
Inhaled epoprostenol	4.72	3.01-7.41
Age	1.54	1.28-1.85
Fio_2_	1.35	1.12-1.63
Respiratory rate	1.25	1.07-1.46
SOFA score	1.23	1.02-1.47
Platelet count	0.79	0.66-0.95

*P* = .04, Hosmer-Lemeshow goodness of fit. SOFA = Sequential Organ Failure Assessment.

## Take-home Points

### Study Question:

Among patients receiving humidified high-flow nasal cannula therapy for SARS-CoV-2 pneumonia, does the administration of inhaled epoprostenol (iEpo) prevent progression to mechanical ventilation?

### Results:

In this multicenter retrospective cohort study, use of iEpo was associated with a higher risk of progression to mechanical ventilation or death in a propensity-matched cohort (hazard ratio, 2.08; 95% CI, 1.73-2.50) and the risk persisted after multilevel modeling (OR, 1.35; 95% CI, 1.23-1.49) and multivariable modeling adjusted for outcome propensity (OR, 4.72; 95% CI, 3.01-7.41).

### Interpretation:

Use of iEpo through high-flow nasal cannula therapy is associated with the need for invasive mechanical ventilation or death.

## ABBREVIATIONS:

HHFNC: humidified high-flow nasal cannula
iEpo: inhaled epoprostenol
IQR: interquartile range
