# Supplementary material for: Use of Inhaled Epoprostenol in Patients With COVID-19 Receiving Humidified, High-Flow Nasal Oxygen Is Associated With Progressive Respiratory Failure
Source: CHEST Crit Care. Author manuscript; Available in PMC 2024 Mar 21. (PMC10956404; doi:10.1016/j.chstcc.2023.100019)
Supplement: 1 [file NIHMS1968792-supplement-1.docx]

### Supplemental information

### **e-Figure 1. Propensity score matching categorized by logit score**

### **
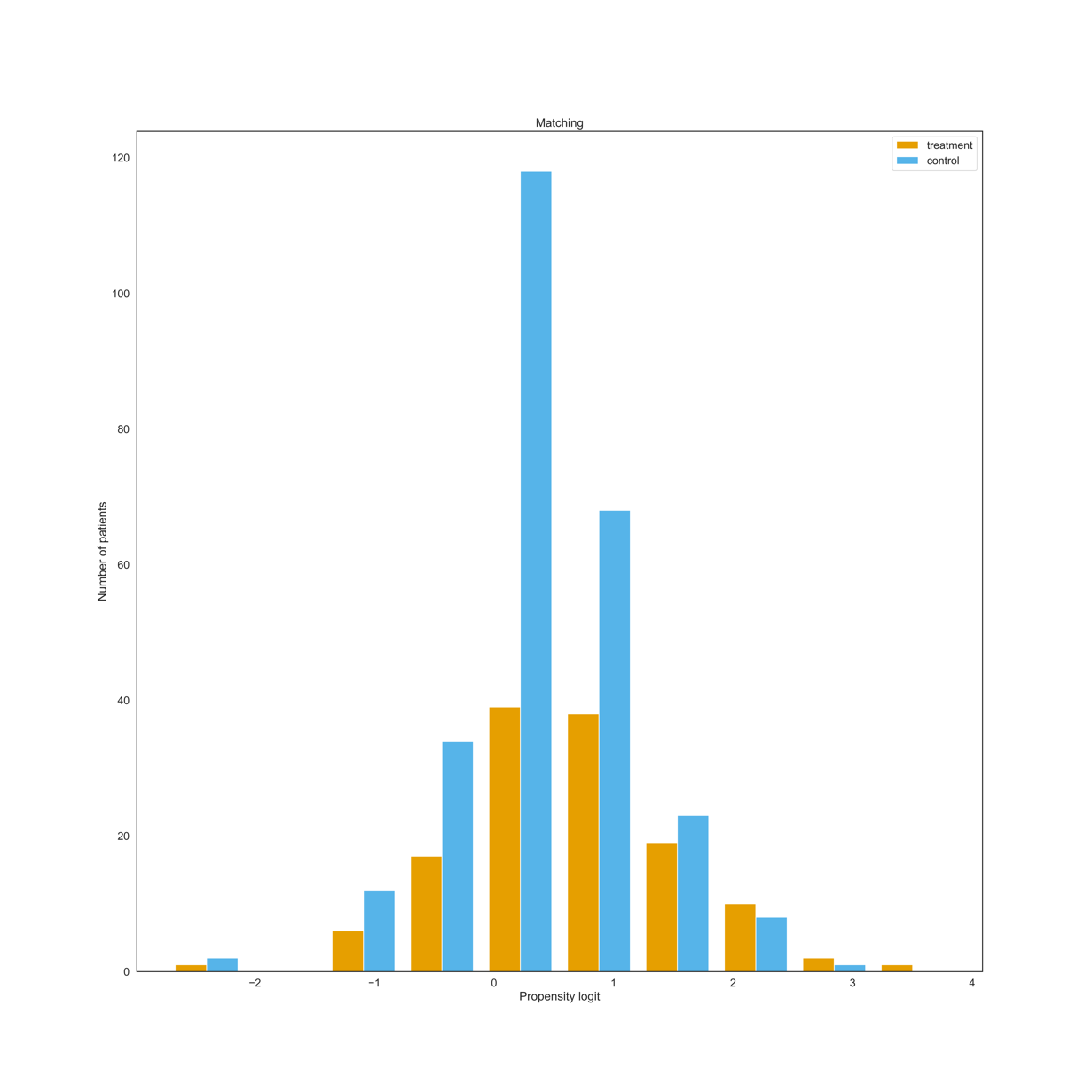
**

### **e-Figure 2. Standardized mean differences across covariates before and after matching**

###
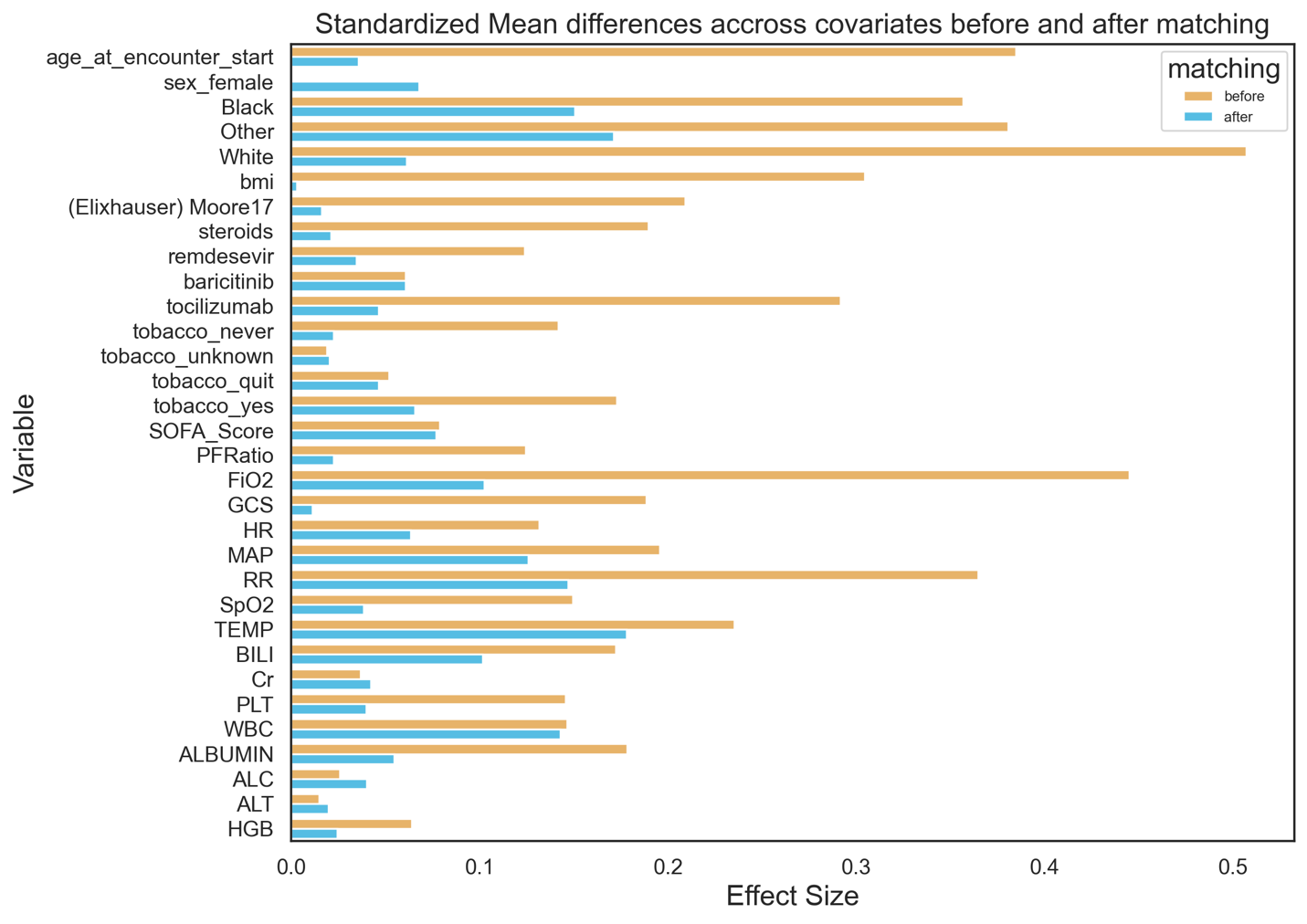


###

### **eFigure-3. QQ Plot for Propensity Score Matched Cohorts**


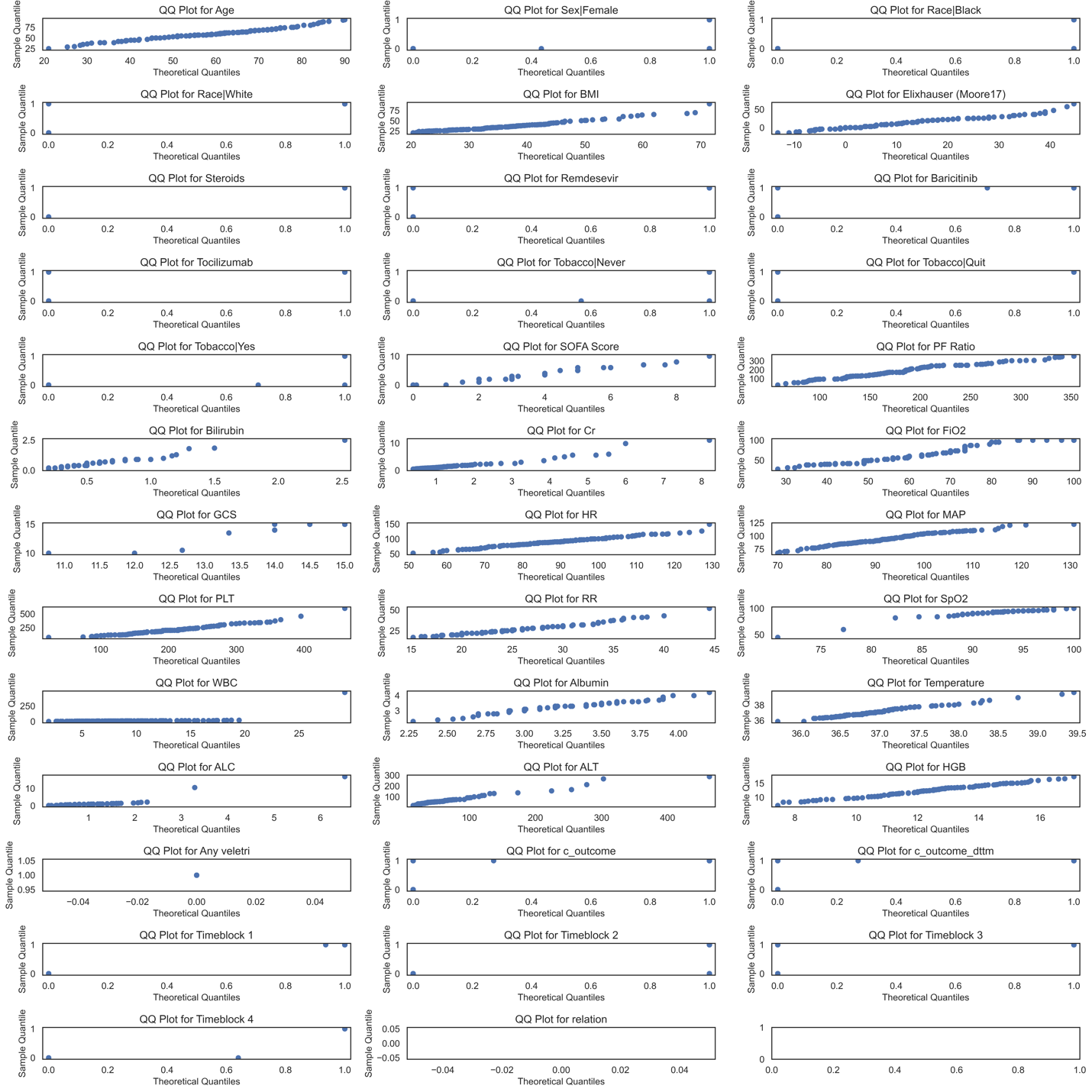


**eMethods 1. Multi-level analysis among propensity score matched patients.**

**Methods:**

All patients matched in a 1:2 ratio were assigned to a relational group that included all patients in that match. Non-categorical data were standardized. For categorical data, 1 item from each category was removed (ex, for patient sex ,only female was retained and male was removed). The model was fit using the statsmodels *mixedlm* function, with the groups set to the relational group of the propensity score matching process. All variables used in the propensity score matching were included in the multi-level model (eTable 1).

**eTable 1. Results of multi-level analysis among propensity score matched patients.**

| Variable | OR (95% CI) |
| --- | --- |
| Any inhaled epoprostenol | 1.35 (1.23-1.49) |
| Any Remdesevir | 0.85 (0.73-0.99) |
| Age at encounter start | 1.12 (1.06-1.19) |
| Peripheral oxygen saturation (SpO2) | 0.95 (0.9-0.99) |
| White blood cell count | 0.95 (0.9-1.0) |
